# Successful Repigmentation of Full-Thickness Wound Healing in Fraser’s Dolphins (*Lagenodelphis hosei*)

**DOI:** 10.3390/ani12121482

**Published:** 2022-06-08

**Authors:** Chen-Yi Su, Hao-Ven Wang, Michael W. Hughes, Tzu-Yu Liu, Cheng-Ming Chuong, Wei-Cheng Yang

**Affiliations:** 1 School of Veterinary Medicine, National Taiwan University, Taipei 10617, Taiwan; angelsu2096@gmail.com; 2 Department of Life Sciences, National Cheng Kung University, Tainan 70101, Taiwan; hvwang@ncku.edu.tw (H.-V.W.); brothansis@yahoo.com.tw (T.-Y.L.); 3 Marine Biology and Cetacean Research Center, National Cheng Kung University, Tainan 70101, Taiwan; 4 International Center for Wound Repair and Regeneration, National Cheng Kung University, Tainan 70101, Taiwan; mwhughes@usc.edu; 5 Institute of Clinical Medicine, National Cheng Kung University, Tainan 70101, Taiwan; 6 Department of Pathology, Keck School of Medicine, University of Southern California, Los Angeles, CA 90007, USA; cmchuong@med.usc.edu

**Keywords:** dolphins, repigmentation, full-thickness wound, melanocytes, melanin

## Abstract

**Simple Summary:**

Scarring with abnormal pigmentation is an undesirable consequence of cutaneous wound healing in humans, which may lead to physiological effects as well as psychological distress. Despite extensive research into cutaneous wound healing and repigmentation, the underlying mechanisms remain largely unknown and there is no reliable and effective treatment to date. Our previous study showed that Fraser’s dolphins (*Lagenodelphis hosei*) have remarkable healing ability to restore skin architecture and pigmentation after full-thickness wounding. In the current study, the association among melanocytes, melanin and skin pigmentation during wound healing in Fraser’s dolphins was investigated. The results showed that the melanocyte density in Fraser’s dolphins was more related to the skin pigmentation than anatomical location and UV exposure, and the timing of melanocyte migration during wound healing in Fraser’s dolphins was different from humans. A better understanding of the mechanisms of successful repigmentation in Fraser’s dolphins will shed light on the development of novel therapies for abnormal pigmentation.

**Abstract:**

Fraser’s dolphins (*Lagenodelphis hosei*) exhibit the capability to restore nearly normal pigmentation after full-thickness wounding. However, the association among melanocytes, melanin and skin pigmentation during wound healing in cetaceans has yet to be addressed. Here, the number of melanocytes and the distribution of melanocytes and melanin in different-colored skin and different wound-healing stages in Fraser’s dolphins were analyzed by using Fontana–Masson staining, immunofluorescence staining and immunohistochemical staining. It was noticed that there was the highest number of melanocytes in dark skin and the lowest number of melanocytes in white skin. The appearance of functional melanocytes and full-melanized neoepidermis was observed in the early stage of wound healing in Fraser’s dolphins. Furthermore, the melanocyte number and skin pigmentation and pattern in healed wounds recovered to a similar condition of unwounded skin. This study provides fundamental knowledge of skin repigmentation in cetaceans for further research, and it will be warranted to elucidate the mechanisms of the replenishment of melanocytes and the regulation of melanocyte activity that contribute to the successful repigmentation in cetacean skin wounds.

## 1. Introduction

Melanocytes are known as melanin-producing cells that provide important physiological functions in photoprotection, pigmentation and immunity [1,2]. Melanocytes produce melanin within membrane-bound organelles termed melanosomes; then, melanosomes are transferred to the surrounding keratinocytes and positioned over the sun-exposed side of nuclei to protect the epidermis from ultraviolet radiation (UVR)-induced DNA damage [3,4]. Melanin is the main determinant of human skin color, although other biomolecules such as carotenoids and hemoglobin can also contribute to skin tone [5]. Skin pigmentation is a sophisticated biological process in which many genes are involved and can be modulated by several intrinsic (e.g., hormonal fluctuations and inflammation) and extrinsic factors (e.g., solar ultraviolet irradiation and environmental pollution) [4,6]. Normal skin pigmentation relies on several critical biological processes, for example, differentiation of melanoblasts into melanocytes, movement of melanocytes from the dermis to the epidermis, production and melanization of melanosomes in the melanocytes, and transfer of melanosomes from the melanocytes to the keratinocytes [7].

In melanocyte-related research, H&E staining and IHC staining are commonly performed for melanocyte identification. It has been demonstrated that melanocytes can be distinguished from keratinocytes by cellular morphology and location with H&E staining [3,8]. Melanocytes reside in the basal layer of the epidermis and have a round to oval nucleus with clear cytoplasm, making it possible to be distinguished from cuboidal to columnar basal cells [9]. IHC staining has been performed in the diagnosis of melanoma [10,11] as well as non-tumor skin samples [12,13]. Several markers have been used for melanocyte identification, for example, S100 protein, human melanoma black 45 (HMB-45), melanoma antigen recognized by T-cells-1 (MART-1, also known as Melan-A), tyrosinase, and microphthalmia-associated transcription factor (MITF) [9,12,13,14,15,16]. S100 proteins constitute a large calcium-binding protein family that regulate cell apoptosis, proliferation, differentiation, migration, energy metabolism, calcium homeostasis, tissue repair and inflammation [17,18,19,20]. S100 proteins are commonly expressed in the cytoplasm and nuclei of melanocytes and other cells [18]. HMB-45 is a marker of premelanosomal glycoprotein gp100 that can recognize immature melanosomes in early-stage melanocytes and reactivated adult melanocytes, but does not detect normal adult melanocytes [21,22,23]. Melan-A is a cytoplasmic protein involved in formation and maturation of melanosome [11] and is expressed in mature melanocytes of normal skin as well as transformed melanocytes of benign and malignant melanocytic neoplasms. [11,16]. Tyrosinase is an important enzyme in the biosynthesis of melanin and is used as a marker of melanocytes, melanocytic naevi and melanoma [10,13,24]. There are several markers for diagnosis of melanoma and melanocytic lesions, but none of them show absolute specificity and sensitivity because of a variety of melanocytic diseases [11]. The combination of several antimelanocyte antibodies, also known as pan-melanocytic cocktail, has been recommended for diagnosis of melanocytic diseases to detect most of the melanocytes [12,13,25].

Skin injury, especially wound healing with severe scarring, may lead to abnormal pigmentation. According to the appearance of the lesional skin, abnormal pigmentation can be categorized into hyperpigmentation, hypopigmentation and a mixed type of pigmentation [26]. Hyperpigmentation usually refers to effects of inflammatory response [27]. Hypopigmentation may result from a lack of functional melanocyte migration into the scar tissue or failure of melanin production and transfer [28,29]. Despite extensive research, the knowledge of repigmentation mechanisms remains limited. Previous studies demonstrated the difference in the timing of melanocyte migration after wounding among animal species. Studies on full-thickness wounds in humans and Duroc pigs showed that melanocytes were absent in the newly formed epidermis until the wound had healed [14,30]. The authors suggested that these findings in pigs supported the hypothesis which melanocytes from unwounded skin migrate into preformed neoepidermis after the wound is fully re-epithelialized. In contrast, melanocytes were present in the migrating epithelial tongue of deep wounds in guinea pigs [31,32], which indicated that melanocytes migrated along with keratinocytes. The aforementioned studies showed that the timing of melanocyte migration might differ among different wound types and different species. It is warranted to investigate the underlying mechanisms contributing to this phenomenon.

The traditional wound-healing animal models, such as mice and rats, are loose-skinned mammals, whereas humans, pigs and cetaceans are tight-skinned mammals (reviewed in [33,34]). Our previous study showed that Fraser’s dolphins (*Lagenodelphis hosei*) exhibit incredible healing ability to restore skin architecture and pigmentation after full-thickness wounding [35]. We suppose that cetaceans may be a novel approach to study wound healing and repigmentation in tight-skinned mammals. There are some studies on melanocyte and melanin in cetacean skin. Melanin granules are present in all epidermal layers of cetacean skin, including the stratum corneum, and the wide distribution of melanin granules was considered as a unique photoprotective trait of cetaceans to adapt to aquatic life [36]. Studies showed that fin whales (*Balaenoptera physalus*), whose skin color is darker compared to blue whales (*Balaenoptera musculus*) and sperm whales (*Physeter macrocephalus*), have a larger quantity of melanocytes and melanin and lower prevalence of UVR-induced skin lesions [8,37]. White-gray skin samples collected from the gray morphism of southern right whales (*Eubalena australis*) had fewer melanocytes than wild-type black skin samples [38]. Another study using skin biopsies from the dorsal surface of cetaceans showed the variances in the melanocyte counts between odontocetes and mysticetes: the numbers of melanocytes varied in odontocetes (bottlenose dolphin (*Tursiops truncatus*), pantropical spotted dolphin (*Stenella attenuata*), and spinner dolphin (*Stenella longirostris*), but not in mysticetes (Bryde’s whale (*Balaenoptera edeni*), fin whale, or humpback whale (*Megaptera novaeangliae*)) [36].

To date, only one study has described repigmentation during skin wound healing in cetaceans [35]. The study showed that melanin and melanocytes were present in the migrating epithelial tongue of full-thickness wounds in Fraser’s dolphins, and the healed wounds restored pigmentation similar to surrounding unwounded skin. However, the association among melanin, melanocyte and skin color during wound healing in cetaceans is unclear, and the mechanisms of successful repigmentation in cetaceans remain largely unknown. The aim of the current study was to investigate the number of melanocytes and the distribution of melanocytes and melanin in different-colored skin and different wound-healing stages in Fraser’s dolphins. This study would shed light on the mechanisms of repigmentation in cetaceans and their implications for regenerative medicine application.

## 2. Materials and Methods

### 2.1. Sample Collection and Preparation

**Figure 1:**
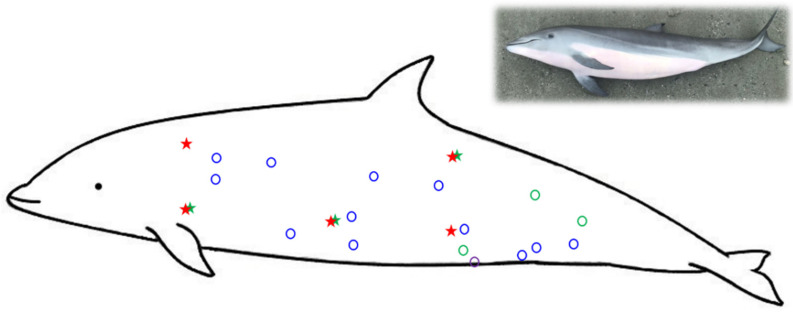
The sampling locations in the current study. ★: normal skin; **○**: wounded skin. Blue marks: animal ID TP20190115; green: IL20191105; red: ML20200807; purple: PT20201109.

The skin samples were collected from four dead stranded Fraser’s dolphins in Taiwan, as detailed in Table 1. All animal procedures were conducted with the approval of the Ocean Conservation Administration (OAC), Taiwan (Permit #1090002352). Body condition and carcass condition were assessed according to previous studies [39,40]. The samples included normal skin and full-thickness wounds caused by cookiecutter shark (*Isistius brasiliensis*) bites. Full-thickness wounds were classified into stage 1 to stage 5, according to a previous study [35]. The sampling locations in the current study are shown in Figure 1. Dark-skin samples were collected from dorsal-lateral, gray skin samples from lateral trunk and peduncle, and white skin samples from ventral side. Tissues were fixed in 10% neutral buffered formalin for 3 days and then embedded in paraffin.

### 2.2. Fontana–Masson Staining

Skin tissues were cut into 4 μm sections for Fontana–Masson staining. Slides were deparaffinized using xylene and rehydrated through graded ethanol, and then subjected to Fontana–Masson staining according to accepted protocol. Images were recorded with a Whited WM100 microscopy (Whited, Taipei, Taiwan).

### 2.3. Immunofluorescence (IF) Staining

Skin tissues were cut into 7 μm sections for IF staining. The sections were baked at 60 °C for one hour. After cooling, the sections were deparaffinized in xylene and rehydrated through graded ethanol. Between following steps, sections were rinsed in TBST (Tris-buffered saline with 0.1% Tween^®^ 20 Detergent) for 5 min 3 times. Antigen retrieval was carried out by using Uni-trieve (Innovex Biosciences, Richmond, CA, USA) at 40 °C overnight. The sections were incubated in blocking buffer at room temperature for 1 h and then incubated in 1st primary antibody, pan-melanocytic cocktail antibody (immunogens: HMB-45, two clones of Melan-A, and tyrosinase; ab733, mouse monoclonal, Abcam, Cambridge, UK. 1:50), at 4 °C overnight. Negative control slides were incubated with PBS only. Following overnight incubation, the sections were incubated in 1% methanol peroxide at 4 °C for 30 min and subsequently incubated with 1st secondary antibody, Cy5^TM^ goat-anti-mouse IgG (A10524, Invitrogen, Carlsbad, CA, USA. 1:100), at room temperature for 1.5 h. The sections were rinsed several times before second stain. The sections were incubated in 2nd primary antibody, S100 antibody (IR504, rabbit polyclonal, Dako, Glostrup, Denmark. Ready-to-use), at 4 °C overnight. Then, the sections were incubated in 2nd secondary antibody, Alexa Fluor^TM^ 594 goat-anti-rabbit IgG (A11012, Invitrogen. 1:200), at room temperature for 1.5 h. Nuclei were counterstained with Hoechst 33342 dye (H3570, Invitrogen. 1:1000) for 5 min and slides were then mounted with aqueous mounting media. Images were recorded with an Olympus IX83 epifluorescence microscopy (Olympus, Tokyo, Japan).

### 2.4. Immunohistochemical (IHC) Staining

Skin tissues were cut into 7 μm sections for IHC staining. The sections were baked at 60 °C for one hour, followed by deparaffinizing and rehydrating. Antigen retrieval was carried out by using Uni-trieve (Innovex Biosciences, Richmond, CA, USA) at 40 °C overnight. Super sensitive^TM^ polymer-HRP detection system (BioGenex Laboratories Inc., Los Angeles, CA, USA) was applied for the immunohistochemical study. Between each following step, the sections were rinsed in TBST (Tris-buffered saline with 0.1% Tween^®^ 20 Detergent). The sections were blocked with Power Block^TM^ universal blocking reagent (BioGenex Laboratories Inc., Los Angeles, CA, USA) at room temperature for 10 min. The primary antibodies applied in the current study included: anti-S100 antibody (IR504, Dako, Glostrup Kommune, Denmark), anti-Melan-A antibody (M7196, mouse monoclonal, Dako. 1:25) and cocktail antibody (ab733, Abcam, Cambridge, UK. 1:50). Primary antibodies were applied overnight at 4 °C. Following overnight incubation, the sections were incubated in 1% methanol peroxide at room temperature for 20 min. Super Enhancer^TM^ reagent was applied at room temperature for 30 min. Polymer-HRP reagent was applied at room temperature for 1 h. The sections were incubated with 3-amino-9-ethylcarbazole (AEC) substrate solution at room temperature for 7 min, followed by counterstaining with Mayer’s hematoxylin for 20 s, and mounted with aqueous mounting media. Images were recorded with a Whited WM100 microscopy (Whited, Taipei, Taiwan).

### 2.5. Cell Counting of Melanocytes

IHC staining with cocktail antibody was performed for the melanocyte count. Positive-stained cells were manually counted along the basement membrane at ×500 viewing field. The length of examined basement membrane was 20–50 mm, depending on the wound size and the pattern of skin color. Results are expressed as number of melanocytes per millimeter.

## 3. Results

### 3.1. Fontana–Masson Staining

**Figure 2:**
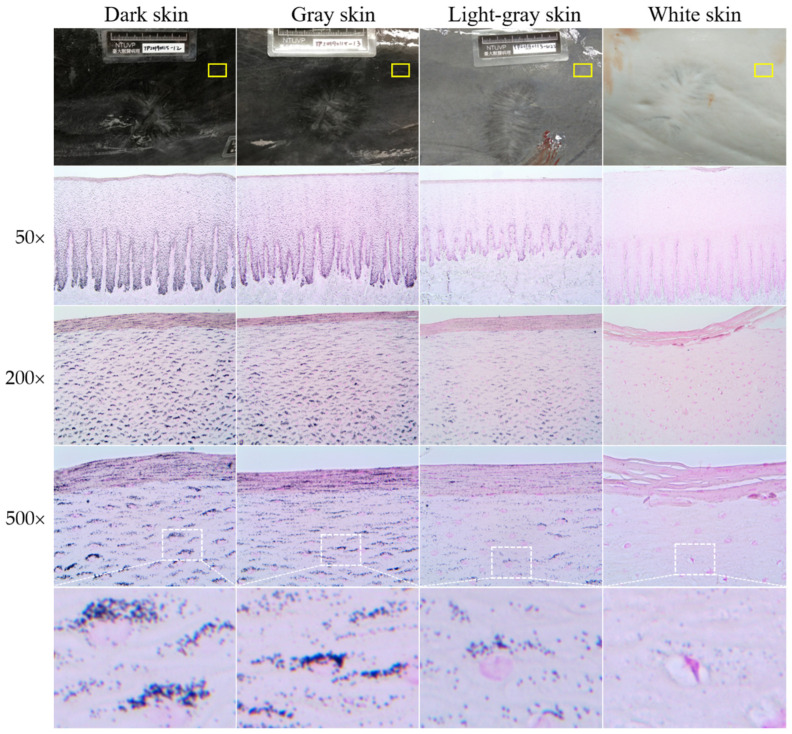
The distribution of melanin in different skin color samples in Fraser’s dolphins. Fontana–Masson staining. All the images were captured from uninjured skin (yellow frame). Melanin granules were present throughout the epidermis, even in the stratum externum of white skin. Apical accumulation of melanin granules above the keratinocyte nucleus was observed in all skin samples. The last row shows the magnified images from the area of the white dotted frame.

**Figure 3:**
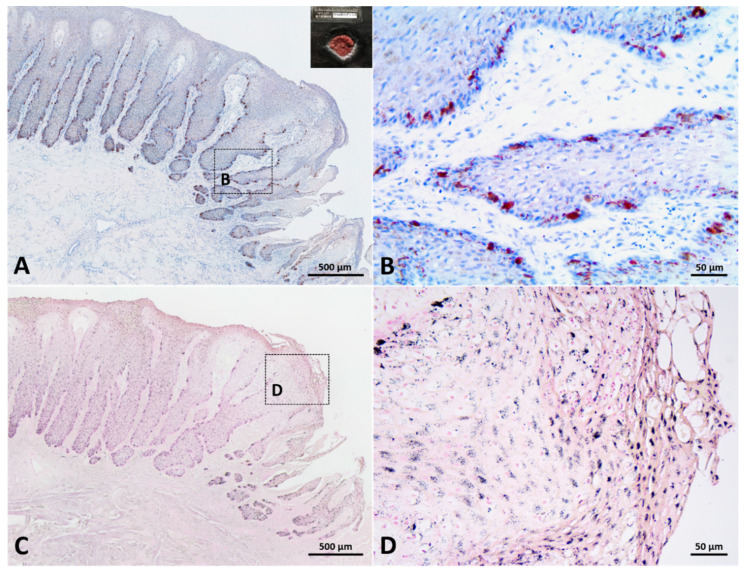
The distribution of melanocytes and melanin in a stage 3 wound collected from dark skin region. (**A**,**B**): Immunohistochemical staining with cocktail antibody. Numerous melanocytes were observed in the basal layer of neoepidermis, including leading edge of migrating epithelial tongue. (**C**,**D**): Fontana–Masson staining. Abundant melanin granules were distributed throughout the neoepidermis, including the stratum externum of leading edge of migrating epithelial tongue.

**Figure 4:**
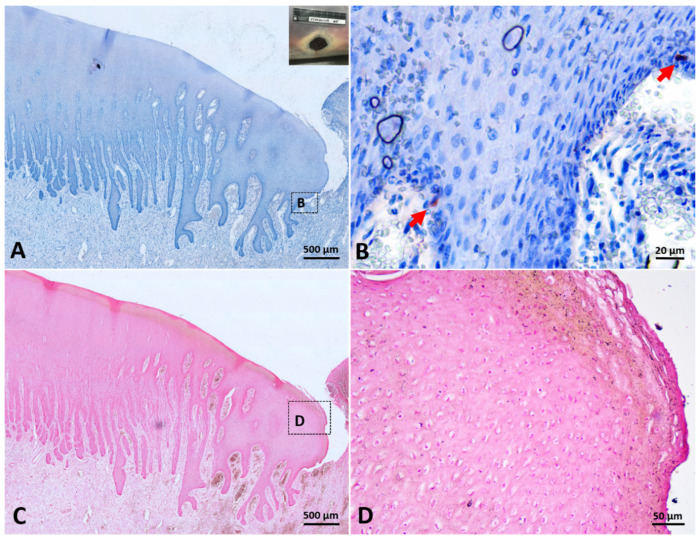
The distribution of melanocytes and melanin in a stage 3 wound collected from white skin region. (**A**,**B**): Immunohistochemical staining with cocktail antibody. Few melanocytes (arrow) were present in the basal layer of neoepidermis, including leading edge of migrating epithelial tongue. (**C**,**D**): Fontana–Masson staining. Scarce melanin granules were distributed throughout the neoepidermis, including the stratum externum of leading edge of migrating epithelial tongue.

More melanin staining was observed in dark skin compared to light-gray and white skin, although staining intensity was not quantified. All the skin samples used in the current study showed melanin granules throughout the epidermis, including the stratum basale, the stratum spinosum, and the stratum externum (Figure 2). Melanin granules were present on the apical side of cell nucleus. In stage 3 wounds (one from the dark skin region and the other from the white skin region), melanin granules were also observed throughout the neoepidermis, including the leading edge of the migrating epithelial tongue (Figure 3 and Figure 4C,D).

### 3.2. Immunofluorescence Staining

**Figure 5:**
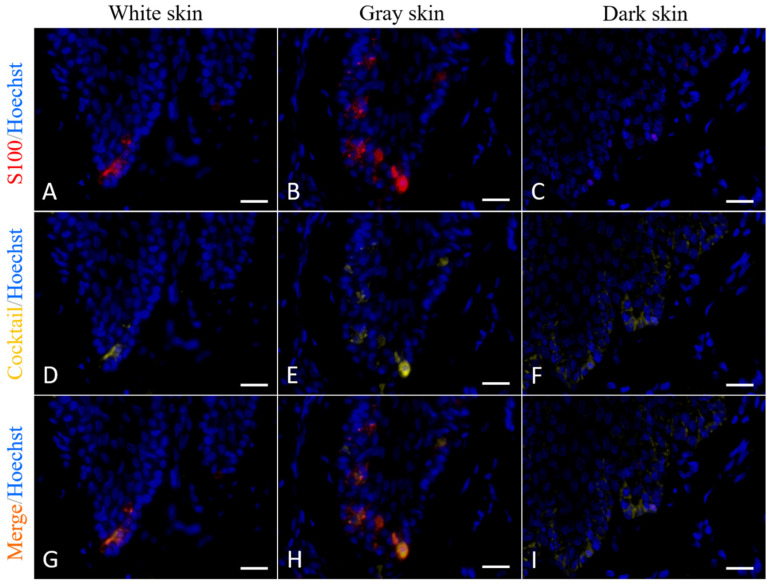
Immunofluorescence staining with anti-S100 antibody and cocktail antibody in normal white/gray/dark skin of Fraser’s dolphins. Signals color: red, S100; yellow, cocktail; blue, Hoechst. Scale bars = 20 μm. (**A**–**C**): IF staining with anti-S100 antibody. (**D**–**F**): IF staining with cocktail antibody. (**G**–**I**): Dual staining with anti-S100 and cocktail antibodies.

In the normal white skin and normal gray skin, S100 and cocktail double-positive cells were observed in the basal layer of the epidermis, and no single-positive cell was found (Figure 5A,B,D,E,G,H). In the normal dark skin, S100 signals were rare and barely visible, while cocktail signals were weak but still visible, and the number of cocktail-positive cells were higher than the number of S100-positive cells (Figure 5C,F,I). The cocktail-positive cells in dark skin were obviously more than those in gray skin and white skin.

### 3.3. Immunohistochemical Staining

**Figure 6:**
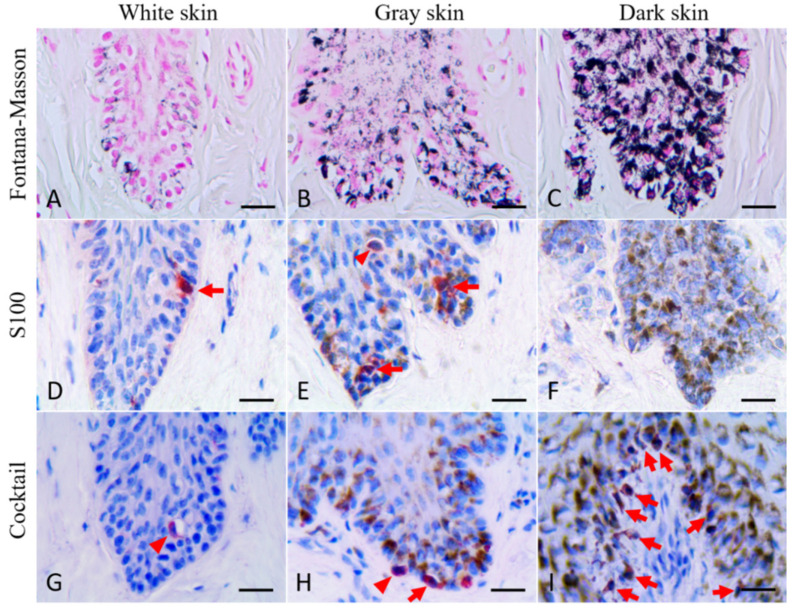
Fontana–Masson staining and immunohistochemical staining with anti-S100 antibody and cocktail antibody in normal white/gray/dark skin of Fraser’s dolphins. Scale bars = 20 μm. (**A**–**C**): Fontana–Masson staining. (**D**–**F**): IHC staining with anti-S100 antibody. (**G**–**I**): IHC staining with cocktail antibody. Some immunoreactive cells had a round to oval nucleus with clear cytoplasm (arrowhead), while some immunoreactive cells exhibited fusiform or dendritic morphology with long cytoplasmic processes (arrow).

**Figure 7:**
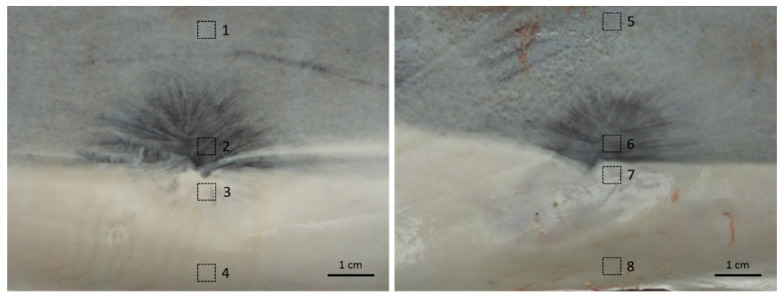
Gross appearance of two full-thickness healed wounds in a Fraser’s dolphin. Site 1 and 5: light-gray unwounded skin; site 2 and 6: gray wounded skin; site 3 and 7: white wounded skin; site 4 and 8: white unwounded skin.

S100-positive cells were observed in the basal layer of white and gray, normal and healed skin (Figure 6D,E). Some S100-positive cells were round or oval, while the others exhibited fusiform or dendritic shape with long cytoplasmic processes. The typical round to oval nucleus with a clear cytoplasm could be observed in some S100-positive cells but not in all S100-positive cells. S100 signals in white skin and gray skin could be identified, but signals in dark skin were too weak to be correctly recognized (Figure 6F). Cocktail-positive cells could be observed in different-colored skin (including white, gray and dark skin) (Figure 6G–I). The morphology of cocktail-positive cells was similar to that of S100-positive cells. Some cocktail-positive melanocytes had a round to oval nucleus with clear cytoplasm, but the others did not. In stage 3 wounds, cocktail-positive cells were observed in the neoepidermis, including the leading edge of the migrating epithelial tongue (Figure 3 and Figure 4A,B). The numbers of melanocytes in different-colored skin were not constant. The highest number of melanocytes was found in dark skin (including wounded, unwounded adjacent skin and normal skin) (14.1 ± 3.9), followed by gray skin (7.5 ± 0.3), light-gray skin (5.6 ± 0.7), and the lowest number of melanocytes in white skin (2.3 ± 1.2) (Table 2). It showed apparent partition of melanocyte numbers among different-colored skin instead of among different healing stages. The skin coloration of two healed full-thickness wounds was similar to that of unwounded skin (Figure 7). The numbers of melanocytes were 5.0 and 5.7 in the light-gray unwounded skin (site 1 and 5), 7.6 and 7.2 in the gray wounded skin (site 2 and 6), 3.2 and 1.6 in the white wounded skin (site 3 and 7), and 3.0 and 1.5 in the white unwounded skin (site 4 and 8). There is no signal observed in IHC staining with anti-Melan-A antibody in the current study despite numerous attempts to modify antigen retrieval and staining protocols.

## 4. Discussion

It has been considered that melanin granules are positioned apically to provide protection against UVR-induced damage [3,36] A recent study showed that cell polarity and the movement of centrosomes and centriolar satellites are implicated in apical distribution of melanin within the human keratinocytes [41]. The previous study in several cetacean species has described the phenomena that melanin granules were arranged apically within keratinocytes [36] although the color of analyzed skin samples was not described. The cellular machinery for apical distribution of melanin in cetacean keratinocytes may be similar to that in human keratinocytes. In the current study, apical accumulation of melanin granules above the keratinocyte nucleus was observed in all skin samples, including white skin. However, photoprotective function of melanin in white skin is supposed to be weak due to the limited quantity of melanin granules in the epidermis. Fraser’s dolphins live in tropical and subtropical oceans, where they receive more direct solar radiation throughout the year. It will be intriguing to investigate photoprotective strategies in those cetacean species with white skin coloration, especially in Chinese white dolphins (*Sousa chinensis*), who live in East and Southeast Asian waters.

Melanocytes in cetacean skin were identified directly with H&E staining or Fontana–Masson staining in previous studies [8,36,37,38]. However, the large amount of melanin accumulated in the basal layer of dark skin in Fraser’s dolphins leads to the difficulty in correctly recognizing the melanocytes with H&E staining or Fontana–Masson staining (Figure 6C). The melanin-bleaching method was tried to solve this problem at the beginning of the present study, but the skin tissues were seriously destroyed after bleaching, making it impossible to count the number of melanocytes with a curled or detached epidermis. Thus, IHC staining was performed for the melanocyte count.

Both IHC staining and IF staining showed a faint expression of S100 in dark skin samples (Figure 5C and Figure 6F). There are two possible explanations: (1) the immunostaining protocol used in the current study might not be optimal in cetacean dark skin despite several modifications having been tried; (2) the expression of S100 proteins in dark skin is lower than that in white and gray skin. The first one is unlikely to be true because IHC and IF signals in white and gray skin samples are clear. The anti-S100 antibody applied in the present study is supposed to react strongly with S100B according to the manufacturer’s instructions. It has been shown that S100B is involved in stimulation of cell proliferation and migration and inhibition of apoptosis and differentiation (reviewed in [42]). We supposed that low expression of S100B may help control an appropriate number of melanocytes in dolphin dark skin. It raises a question whether the expression of S100B increases during wound healing to promote melanocyte proliferation. Further research is needed to elucidate the role of S100 proteins in melanocyte regulation in different skin colorations and conditions in dolphins.

In the current study, we applied a pan-melanocytic cocktail in order to detect all melanocytes in different developmental stages. It was noticed that some cocktail-positive cells did not have typical clear cytoplasm around the nucleus, indicating that IHC staining with cocktail antibody could detect more melanocytes than H&E staining does. Furthermore, the results of IF staining with cocktail antibody in white and gray skin were consistent with the results of IF staining with S100 antibody (Figure 5G,H). Taken together, it is very likely that all developmental stages of melanocytes in Fraser’s dolphin skin were detected by applying cocktail antibody. So, the melanocyte count was based on the results of IHC staining with cocktail antibody. It showed that the melanocyte density in the studied samples in Fraser’s dolphins was uneven, which means that darker skin in the normal and wounded samples had more melanocytes and vice versa. In contrast, there was no significant difference in the number of melanocytes between hyperpigmented and hypopigmented scars in red Duroc pigs [21]. Because the dark skin samples in the current study were collected from dorsal-lateral sites and white skin samples were collected from ventral sites, it was supposed that the uneven melanocyte distribution is related to anatomical location or UV exposure. It is known that the precursors of melanocytes are derived from neural crest and migrate along a dorsal-lateral pathway during development [43]. A recent study showed that the human melanocyte densities in the UV-exposed parts were obviously higher than those in the unexposed parts [44]. However, it is striking that healed wounds in the junction of gray and white skin (Figure 7) demonstrated that the melanocyte density differs markedly within a small region. It indicates that the melanocyte density of Fraser’s dolphins is more correlated with skin pigmentation rather than anatomical location and UV exposure. It is known that human skin pigmentation is determined by the quantity of melanin; eumelanin to pheomelanin ratio (EPR); and the number, size and distribution of melanosomes, whereas the number of melanocytes remains relatively constant among different ethnicities [3]. Further studies on melanogenesis, EPR and melanosome characteristics in dolphin skin will lead to a better understanding of the determinants of dolphin skin pigmentation.

Of note, melanocytes and melanin granules were observed in the migrating epithelial tongue in dark and white skin in Fraser’s dolphins. This finding was similar to what had been reported in guinea pigs [31,32], but differs from what has been known in humans [30] and pigs [14]. In the full-thickness wound, melanocytes from the surrounding unwounded skin were considered as the only available source of melanocytes for repigmentation [27]. However, human dermal stem cells have been supposed to be capable of homing to the epidermis to differentiate into functional melanocytes, suggesting that dermal stem cells may be another source of epidermal melanocytes [45,46]. It raises a question of whether melanocytes in the neoepidermis of cetacean skin wounds come from the unwounded area around the wound or partially originate from dermal stem cells. Moreover, the appearance of melanocytes in the early stage of wound healing implies its important role in cetacean wound healing. Melanocytes exhibit not only photoprotective function but immune properties. It has been known that human skin melanocytes express several immunologically relevant surface molecules and participate in immune responses in many ways, for example, cytokine and chemokine production [47,48], phagocytosis and antigen presentation [49] and immunomodulation [50,51]. Little is known about the immune function of melanocytes in cetacean skin, and new studies are warranted in an attempt to detail the possible important role of melanocytes during wound healing in cetaceans.

The current study showed the early appearance of functional melanocytes and the full-melanized neoepidermis during full-thickness wound healing in Fraser’s dolphins, and, the most intriguing finding, the melanocyte number and skin pigmentation in healed wounds recovered to a similar condition of unwounded skin. Successful repigmentation after skin injury depends on multiple factors, such as migration of functional melanocytes into the neoepidermis, synthesis of melanin and transfer of melanin to adjacent keratinocytes [14]. To date, it remains unclear whether the melanocytes in the dolphin neoepidermis originate from the surrounding unwounded skin or differentiate from dermal stem cells. It is also unknown which microenvironmental factors attract melanocytes to the wounded area and regulate the production of melanin at the appropriate level. Future research may focus on the source of melanocytes for repigmentation and the factors which regulate melanocyte migration, proliferation and activity in dolphin skin during wound healing. These in-depth investigations will advance our understanding of the mechanisms of successful repigmentation in Fraser’s dolphins.

## Figures and Tables

**Table 1:** The details of sampled dolphins in the current study.

Animal ID	Gender	Age	Body Condition	Carcass Condition
TP20190115	Male	Adult	Thin	Freshly dead
IL20191105	Male	Subadult	Thin	Freshly dead
ML20200807	Male	Adult	Thin	Moderate decomposition
PT20201109	Male	Subadult	Thin	Freshly dead

**Table 2:** Cell counting of melanocytes.

Sample No.	Skin Condition	Wounded Skin		Unwounded Skin	
		Count	Color	Count	Color
1	Stage 3 wound	17.8	Dark	20.6	Dark
2	Stage 4 wound	8.8	Dark	9.6	Dark
3	Stage 5 wound	12.2	Dark	10.6	Dark
4	Stage 4 wound	7.4	Gray	12.7	Dark
5	Stage 5 wound	7.0	Gray	7.7	Gray
6	Stage 5 wound	7.8	Gray	7.7	Gray
7	Stage 4 wound	7.6	Gray	5.0	Light gray
8	Stage 5 wound	7.2	Gray	5.7	Light gray
9	Stage 4 wound	4.2	Light gray	6.4	Light gray
10	Stage 4 wound	5.5	Light gray	6.6	Light gray
11	Stage 4 wound	5.3	Light gray	5.5	Light gray
12	Stage 5 wound	6.1	Light gray	5.3	Light gray
13	Stage 3 wound	2.3	White	4.3	White
14	Stage 4 wound	3.2	White	3.0	White
15	Stage 4 wound	0.2	White	4.0	White
16	Stage 4 wound	0.6	White	1.7	White
17	Stage 5 wound	1.6	White	1.5	White
18	Stage 5 wound	2.1	White	2.9	White
19	Normal skin	NA	NA	15.1	Dark
20	Normal skin	NA	NA	16.9	Dark
21	Normal skin	NA	NA	16.8	Dark
22	Normal skin	NA	NA	6.4	Light gray
23	Normal skin	NA	NA	1.3	White
24	Normal skin	NA	NA	1.5	White
25	Normal skin	NA	NA	3.6	White
26	Normal skin	NA	NA	3.3	White

Wounded skin at stage 3 = migrating epithelial tongue; Wounded skin at stage 4 and 5 = neoepidermis of wound center; Unwounded skin at stage 3, 4 and 5 = unwounded skin adjacent to the wound, approximately 2–3 cm away from the wound edge; Normal skin = normal skin far away from any wound; Count = number of melanocytes per millimeter.

## Data Availability

Not applicable.
